# Supplementary material for: Predicting and mapping soil available water capacity in Korea
Source: PeerJ. 2013 Apr 23;1:e71. doi: 10.7717/peerj.71 (PMC3642705; doi:10.7717/peerj.71)
Supplement: Tables S1-S5 [file peerj-01-71-s001.docx]

T1. Statistics of the Korean soil database used in this study.

|  | n | Min | Max | Median | Mean | Std.dev |
| --- | --- | --- | --- | --- | --- | --- |
| Clay (g/100 g) | 1598 | 0 | 83 | 20 | 21.7 | 12.5 |
| Sand (g/100 g) | 1598 | 0 | 98 | 31 | 35.3 | 25.1 |
| OM  (g/kg) | 1598 | 0 | 55 | 1.2 | 2.15 | 3.51 |
| Bulk density  (g/cm^3^) | 108 | 0.36 | 1.85 | 1.29 | 1.29 | 0.22 |
| w at -10 kPa  (g/100 g) | 1209 | 1.4 | 117 | 37 | 37.4 | 13.8 |
| w at -33 kPa  (g/100 g) | 1452 | 0.9 | 88 | 30 | 29.6 | 12.00 |
| w at -1500 kPa  (g/100 g) | 1443 | 0.4 | 59 | 12 | 13.5 | 7.49 |

T2. Statistics of the 301 soil samples from the US National Soil Characterization database used to develop the calibration function for field capacity.

|  | Mean | Std. dev | Median | Min | Max |
| --- | --- | --- | --- | --- | --- |
| w_10_ clod (g/100g) | 24.97 | 10.12 | 24.30 | 1.8 | 52.0 |
| w_10_ disturbed (g/100g) | 22.70 | 8.80 | 22.60 | 2.5 | 51.4 |
| Bulk density  (g/cm^3^) | 1.44 | 0.22 | 1.48 | 0.88 | 1.97 |
| Clay  (g/100g) | 28.51 | 18.36 | 27.60 | 0.0 | 76.70 |
| Sand  (g/100g) | 38.50 | 30.71 | 29.10 | 0.0 | 98.30 |

T3. Statistics of the 9 soil samples used to validate the prediction of field capacity.

| Soil Series | Depth | Land Use | No. samples | Measured water content at -10kPa (%) | Predicted water content at -10kPa(%) |
| --- | --- | --- | --- | --- | --- |
| Chahang | 0-20 | Upland | 4 | 40 | 44.6 |
| Chahang | 60-80 | Upland | 2 | 29 | 31.9 |
| Jeonnam | 0-20 | Grassland | 2 | 43 | 45.1 |
| Weongog | 0-20 | Grassland | 2 | 43 | 36.6 |
| Songjeong | 0-20 | Upland | 2 | 29 | 36.2 |
| Samgag | 0-20 | Forest | 2 | 21 | 32.0 |
| Gopyeong | 0-20 | Upland | 1 | 42 | 46.4 |
| Gopyeong | 20-40 | Upland | 1 | 37 | 39.4 |
| Jungdong | 0-20 | Upland | 2 | 29 | 39.3 |

T4. Amount of profile available water capacity (PAWC) by land use in Korea.

|  | Area (km^2^) | PAWC (mm) | |
| --- | --- | --- | --- |
|  |  | Mean | Std. dev. |
| Forest | 64,641 | 127 | 72 |
| Paddy field | 11,875 | 231 | 61 |
| Upland field | 7,809 | 189 | 64 |
| Orchard | 548 | 180 | 38 |

T5. Amount of profile available water capacity (PAWC) by topography in Korea.

| Topography | Area(km^2^) | Mean AWC (mm) | Std. dev  (mm) |
| --- | --- | --- | --- |
| Valley plain | 11,026 | 217 | 35 |
| Hill | 11,963 | 177 | 68 |
| Cinder cone | 111 | 140 | 39 |
| Mountain foot sloped land | 8,216 | 180 | 34 |
| Mountainous Land | 49,584 | 136 | 71 |
| Alluvial fan | 2,909 | 165 | 58 |
| Lava flow land | 1,413 | 141 | 77 |
| Alluvial plain | 5,064 | 208 | 88 |
| Fluvio-marine plain | 3,473 | 235 | 84 |
| Diluvial terrace | 1,091 | 213 | 23 |

T5.

Examples of typical soil profiles for paddy field are shown in Figure F2, upland soils in Figure F3, forest in Figure F4, and volcanic ash soils in Figure F5. PAWC of soils under forests (F4) are limited by shallower depth compared the soils under paddy and upland. This is due to the topographic differences, soils under forests are mainly in areas with high slope, such as hills or rolling to mountain, vulnerable to erosion in monsoon season, resulting in shallow depth. On the contrary, paddy soils are located in the lowland, with depositional materials resulting in deeper soil. Besides, soil series with relatively higher silt contents and distributed in plains, Mangyeong, Jeonbug, and Ihyeon (F2) has higher PAWC than other soil series. In forest (F4), the Daesan series with fine silty textural family also shows higher PAWC compared to other soil series. Korean paddy soil distributed lowland has relatively higher silt content compared to forest and upland soils, resulting in higher PAWC (NIAST, 1992). The volcanic ash soils (Andisols) are mainly distributed in inlands in mountain regions or lava terraces. The water retention data (F5), has no clear pattern with organic matter content (Heugag > Ara > Hyangmog). This is probably due to different gravel content and bulk densities. Low bulk densities of the Andisols can offset the increasing effect of organic matter on water retention, in volumetric basis.
