# Supplementary material for: Predicting and mapping soil available water capacity in Korea
Source: PeerJ. 2013 Apr 23;1:e71. doi: 10.7717/peerj.71 (PMC3642705; doi:10.7717/peerj.71)
Supplement: Figures S1-S5 [file peerj-01-71-s002.pdf]

F1

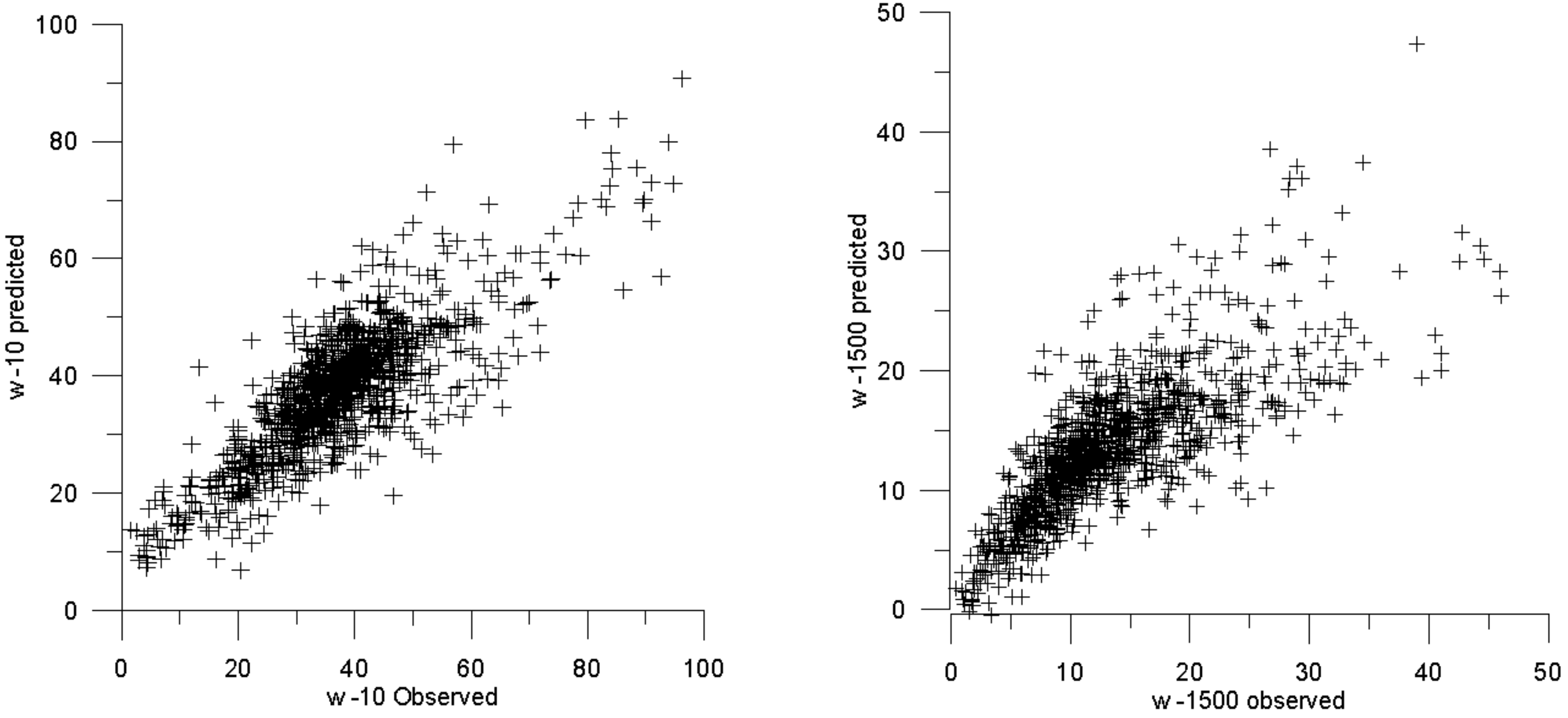

100
80
60
40
20
0
w -10 predicted
0
20
40
60
80
100
w -10 Observed
50
40
30
20
10
0
w -1500 predicted
0
10
20
30
40
50
w -1500 observed

F2

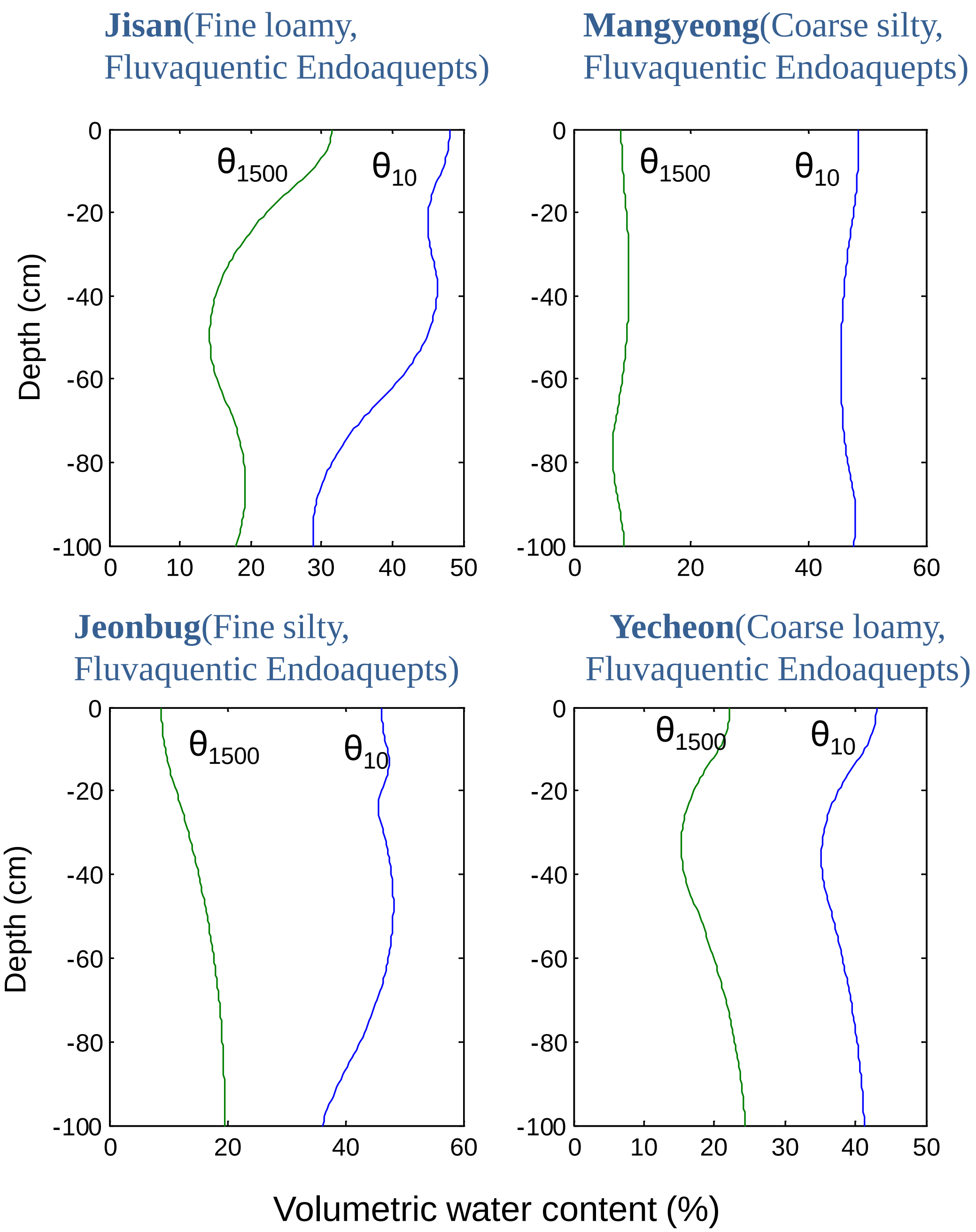

Jisan(Fine loamy, Fluvaquentic Endoaquepts)
Mangyeong(Coarse silty, Fluvaquentic Endoaquepts)
Jeonbug(Fine silty, Fluvaquentic Endoaquepts)
Yecheon(Coarse loamy, Fluvaquentic Endoaquepts)
θ1500
θ10
Depth (cm)
Volumetric water content (%)

F3

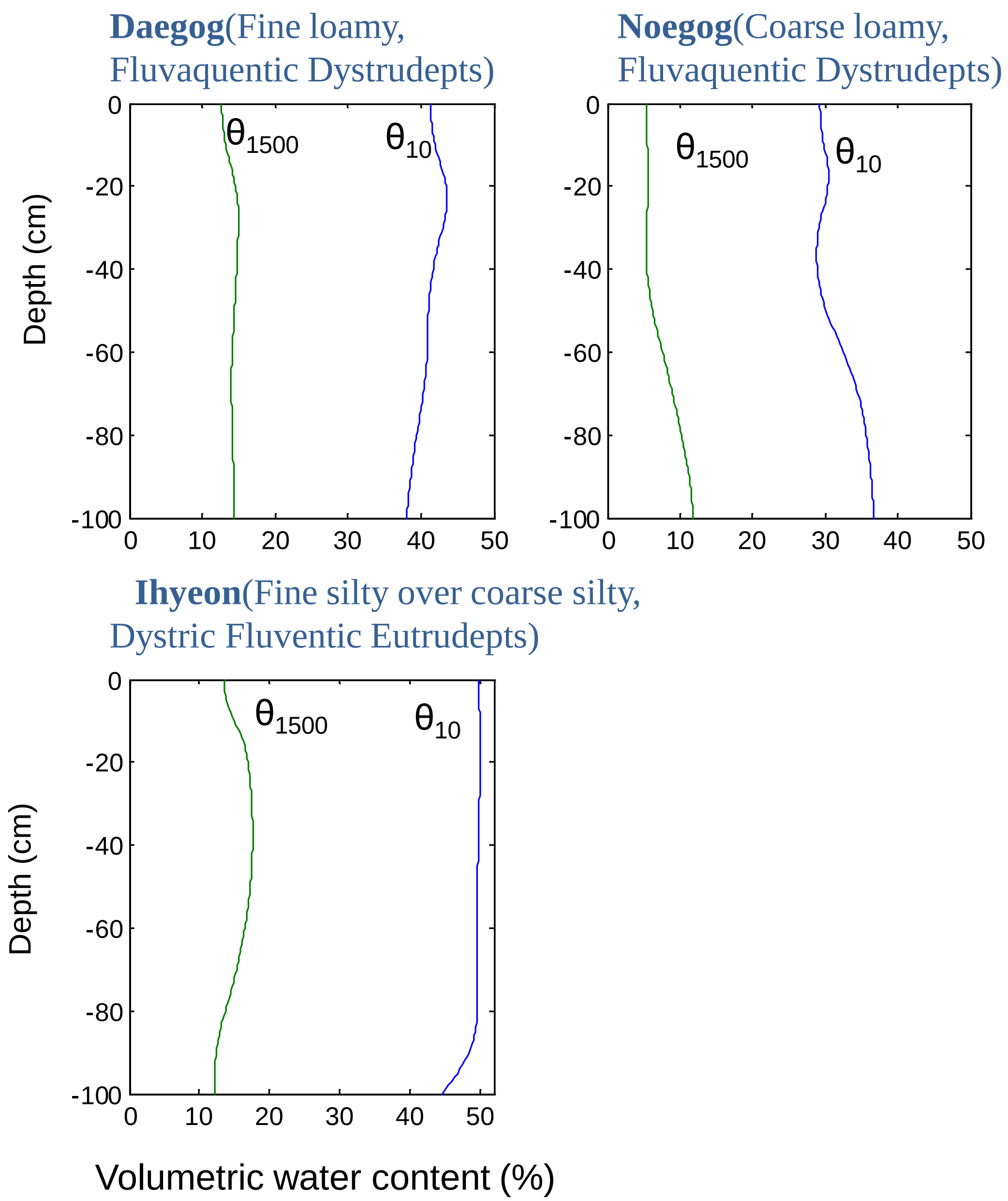
Daegog(Fine loamy, Fluvaquentic Dystrudepts)
Noegog(Coarse loamy, Fluvaquentic Dystrudepts)
Ihyeon(Fine silty over coarse silty, Dystric Fluventic Eutrudepts)
θ_{1500}
θ_{10}
Depth (cm)
0
-20
-40
-60
-80
-100
0
10
20
30
40
50
Volumetric water content (%)

F4

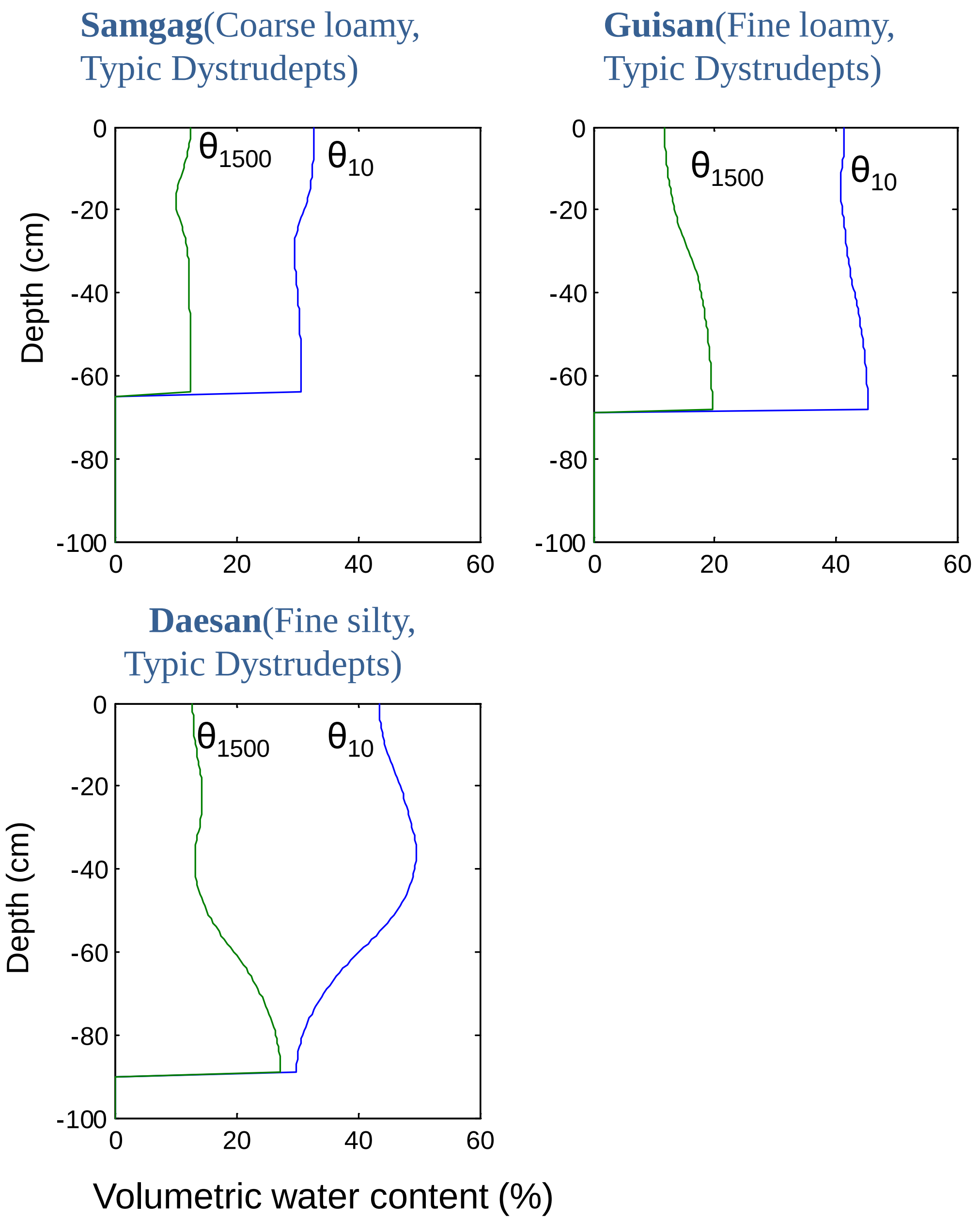

Samgag(Coarse loamy, Typic Dystrudepts)
Guisan(Fine loamy, Typic Dystrudepts)
Daesan(Fine silty, Typic Dystrudepts)
θ_{1500}
θ_{10}
Depth (cm)
Volumetric water content (%)
0
-20
-40
-60
-80
-100
0
20
40
60

F5

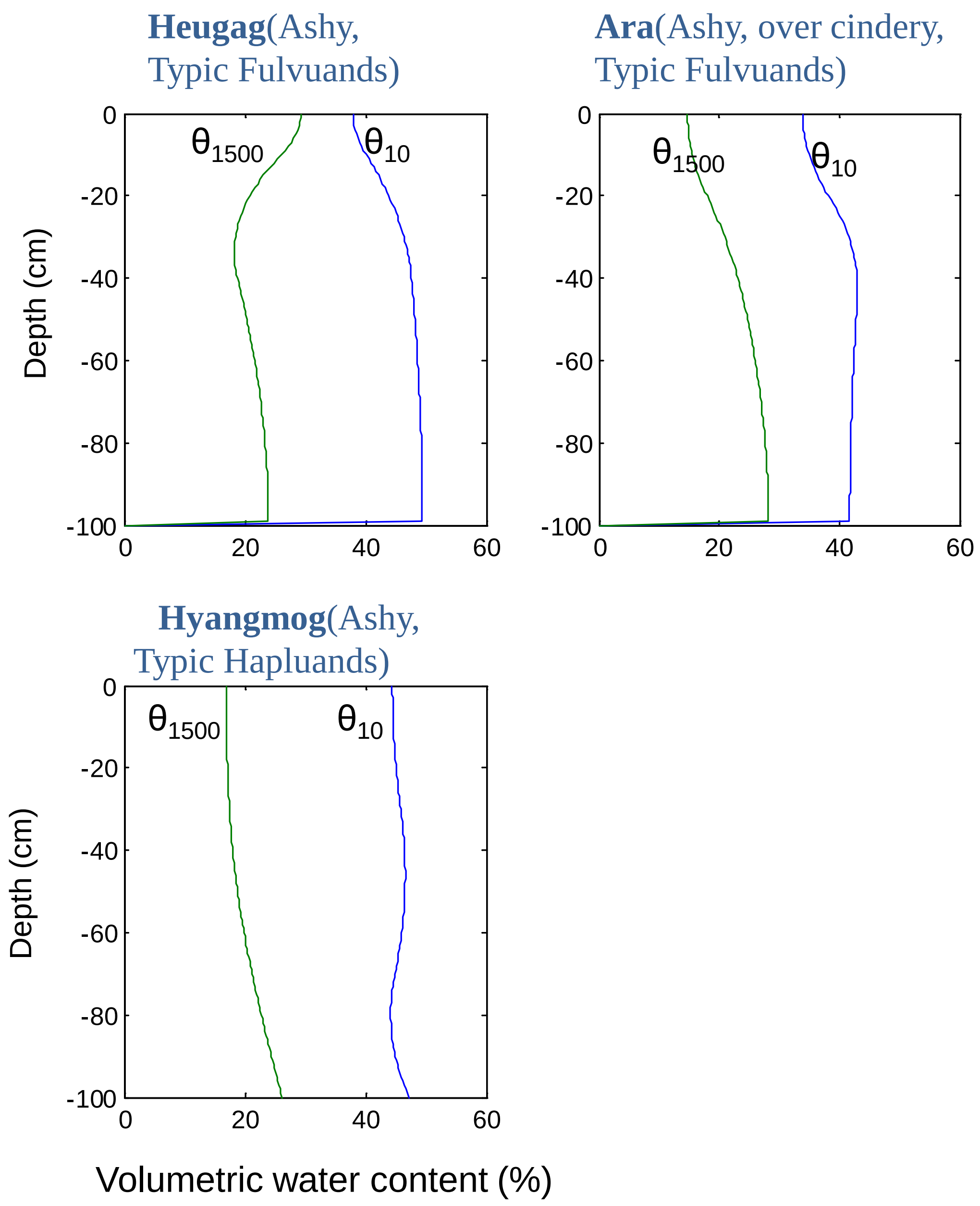

Heugag(Ashy, Typic Fulvuands)
Ara(Ashy, over cindery, Typic Fulvuands)
Hyangmog(Ashy, Typic Hapluands)
θ1500
θ10
Depth (cm)
0
-20
-40
-60
-80
-100
0
20
40
60
Volumetric water content (%)
